# The Role of Hypoxia-Inducible Factor Isoforms in Breast Cancer and Perspectives on Their Inhibition in Therapy

**DOI:** 10.3390/cancers14184518

**Published:** 2022-09-17

**Authors:** Karolina Kozal, Anna Krześlak

**Affiliations:** Department of Cytobiochemistry, Faculty of Biology and Environmental Protection, University of Lodz, Pomorska 141/143, 90-236 Lodz, Poland

**Keywords:** breast cancer, hypoxia, HIF-1α, HIF-2α, metabolism, metastasis, angiogenesis

## Abstract

**Simple Summary:**

In many types of cancers, the activity of the hypoxia-inducible factors enhances hallmarks such as suppression of the immune response, altered metabolism, angiogenesis, invasion, metastasis, and more. As a result of observing these features, HIFs became attractive targets in designing anticancer therapy. The lack of effective breast treatment based on HIFs inhibitors and the elusive role of those factors in this type of cancer raises the concern wheter targeting hypoxia-inducible factors is the right path. Results of the study on breast cancer cell lines suggest the need to consider aspects like HIF-1α versus HIF-2α isoforms inhibition, double versus singular isoform inhibition, different hormone receptors status, metastases, and perhaps different not yet investigated issues. In other words, targeting hypoxia-inducible factors in breast cancers should be preceded by a better understanding of their role in this type of cancer. The aim of this paper is to review the role, functions, and perspectives on hypoxia-inducible factors inhibition in breast cancer.

**Abstract:**

Hypoxia is a common feature associated with many types of cancer. The activity of the hypoxia-inducible factors (HIFs), the critical element of response and adaptation to hypoxia, enhances cancer hallmarks such as suppression of the immune response, altered metabolism, angiogenesis, invasion, metastasis, and more. The HIF-1α and HIF-2α isoforms show similar regulation characteristics, although they are active in different types of hypoxia and can show different or even opposite effects. Breast cancers present several unique ways of non-canonical hypoxia-inducible factors activity induction, not limited to the hypoxia itself. This review summarizes different effects of HIFs activation in breast cancer, where areas such as metabolism, evasion of the immune response, cell survival and death, angiogenesis, invasion, metastasis, cancer stem cells, and hormone receptors status have been covered. The differences between HIF-1α and HIF-2α activity and their impacts are given special attention. The paper also discusses perspectives on using hypoxia-inducible factors as targets in anticancer therapy, given current knowledge acquired in molecular studies.

## 1. Hypoxia in Normal and Cancer Cells

By simple definition, hypoxia is a state that results from an insufficient oxygen supply compared to the physiological demand. It can be divided into physiological hypoxia, which is quickly inverted by increased blood flow and proteome changes induced by hypoxia-inducible factors, and pathological hypoxia, which leads to compromised biological function. Physiological hypoxia occurs during embryonic development, during intense physical exercise, and at high altitudes. Pathological hypoxia can be associated with pulmonary diseases, anemia, incorrect methemoglobin formation, poisoning, or reduced tissue perfusion, which is also present in solid tumors [1].

Hypoxia can be defined through an array of other oxygenation states. On one end of the scale, there’s anoxia which is the state of a complete lack of O_2_ in the tissue. A solid definition is harder to establish when the other end of the scale is considered. In opposition to hypoxia, normoxia is oftentimes mentioned. The term normoxia corresponds to atmospheric oxygen pressure which is usually provided for in vitro cultures. The culture’s oxygenation conditions, which are generally approximately 21% pO_2_, are not representative compared to the physiological state of the cells. Tissues have various balances of the gas mixture depending on their vascularization and physiological demand. Oxygenation ranges from 19.7%, observed in inspired air in the trachea, to 1% in the skin’s superficial areas. It is, therefore, impossible to define hypoxia based on the concrete boundaries of the oxygen pressure percentages. In conclusion, hypoxia comprehension has raised the need to specify an additional term—physioxia, which corresponds to the physiological condition of oxygen pressure observed in normal tissue. In the face of the realization of heterogeneous oxygenation, hypoxia can be defined as the level below physioxia [2].

The oxygenation level in cancer cells tends to be lower than in their respective tissues [2]. Höckel and Vaupel have proposed to define hypoxia in tumors by the level of the pO_2_ drop that causes a reduction in O_2_ consumption and ATP production. Hypoxia can also be tracked by inducing specific proteome changes that allow cancer cells to adapt to resource deprivation. These changes include alterations in the activity of glycolytic enzymes and elements involved in angiogenesis, invasiveness, and resistance [1]. Another way of defining hypoxia in cancer is by determining the critical oxygenation point which, as proposed in the work of Carreau et al., is 8–10 mmHg. However, it is crucial to understand that tumors have an uneven distribution of hypoxic and anoxic areas and oxygenation levels, just like in normal tissue, and it can range according to the cancer cell’s origin [2].

The state of hypoxia can be distinguished between acute hypoxia, which results from perfusion-limited oxygen delivery, and chronic hypoxia, which develops from diffusion-limited oxygenation [3]. Chronic hypoxia in tumors can be caused by the poor quality of the vasculature, worsened diffusion geometry, and disturbed circulation, and it can be deliberately sustained for the benefit of the cancer cells [1]. In normal cells, hypoxia occurrence leads to a reduction in protein synthesis, decreased proliferation, and, eventually, death by apoptosis or necrosis. In cancer cells, hypoxia promotes cell proliferation and tumor progression, which develops a more aggressive phenotype [3,4]. Insufficient oxygenation indirectly damages DNA, inducing mutation, breaks, oxidative base damages, and over-replication. DNA damages result from hypoxia-mediated disturbances in the transport and current of electrons, which induce ROS (reactive oxygen species) production. Hypoxia also promotes polyploidy, which results from replication of the genome without dividing cells. Changes induced in genes incline adaptation and metastasis of cancer cells [4]. Hypoxia in cancer cells favors the selection of more hypoxia-resistant cells that tend to be more malignant. Oxygenation deficiency also promotes radiation and anti-cancer drug resistance; it may also reduce the effectiveness of photodynamic therapy [1]. The occurrence of hypoxia disturbs the delivery and activity of many anticancer drugs; it also decreases the efficiency of pH-dependent compounds and alkylating agents [4].

## 2. Hypoxia-Inducible Factors: Characteristics and Functions

The crucial element of the hypoxia response and adaptation is the activity of the transcriptional factor called the hypoxia-inducible factor. It contains two subunits: the oxygen-labile alpha subunit (which has three isoforms: 1α, 2α, and 3α) and the beta subunit, also called ARNT (Aryl Hydrocarbon Receptor Nuclear Translocator). HIFs belong to the bHLH-PAS protein family, and both subunits contain basic helix-loop-helix-PAS domains responsible for complexing with each other and binding DNA [5]. Under physioxic conditions, the alpha subunits have a half-life of approximately five minutes, after which they undergo proteasomal degradation. The process of the alpha subunit disposal is orchestrated mainly by proteins belonging to the superfamily of 2-oxoglutarate-depended dioxygenases—prolyl hydroxylases (PHDs) [4]. Prolyl hydroxylation occurs on specific sites in the oxygen-dependent domain: in HIF-1α on prolines P402 and P564, in HIF-2α on prolines P405 and P531, and in HIF-3α on proline P492 [6]. Hydroxylation of the prolyl residues results in increased affinity of the subunit to von Hippel-Lindau tumor suppressor protein. After hydroxylation, the VHLp, which is endowed with E3 ubiquitin ligase activity, recognizes modified HIF-1α and polyubiquitinates it directing the subunit to the proteasomal degradation [4]. Hydroxylation sites can be modified independently but usually get hydroxylated simultaneously for redundancy [7]. The second mechanism of the HIF-α regulation in physioxia is hydroxylation of the asparaginyl residue catalyzed by the factor inhibiting HIF (FIH). Modification of asparagine results in blocking the hypoxia-inducible factor co-activators binding [5]. The asparaginyl hydroxylation occurs in the C-terminal transactivation domain (C-TAD) domain on N803 in HIF-1α and on N851 in HIF-2α. The HIF-3α does not contain the C-TAD domain; hence the FIH activity does not affect it [6]. The 3α isoform is the least known, so the vast majority of the following text will describe the characteristics and differences of the HIF-1α and HIF-2α isoforms. The HIF-3α isoform activity is probably another mechanism of the HIF-1α and HIF-2α isoforms regulation. It is believed that some splicing variants of the 3α isoform, for example, the inhibitor PAS domain (IPAS), negatively regulate the activity of other isoforms of the hypoxia-inducible factor [8].

The HIF-1α and HIF-2α isoforms show similar regulation characteristics, although they are active in different types of hypoxia. In molecule characteristics, those isoforms share approximately 48% amino acid sequence identity [8]. The HIF-2α, also called the endothelial PAS domain protein (EPAS1), differs from the HIF-1α subunit mainly in the N-terminal transactivation domain (N-TAD), while C-TAD domains share 67% of the similarities [9]. Under insufficient oxygenation, prolyl hydroxylases lose activity due to the lack of the O_2_ substrate, which results in the stabilization of the alpha subunit [5]. The stabilized alpha subunit complexes with the constitutively expressed beta subunit and creates an active heterodimer. The hypoxia-inducible factor complex then can bind to the hypoxia response elements (HREs) in DNA with the help of recruited co-activators CBP/p300. The active heterodimer binds to promoters or enhancers of target genes [10]. Recent studies discovered 2450 potential transcription target genes of the hypoxia-inducible factor [11], but, usually, the number of downstream genes that are regulated and can be observed ranges from 100 to 150 targets [4,10]. The HIF-1α and HIF-2α share a significant portion of target genes, but some targets are unique only to one of them [4].

The HIF-1α is stabilized due to the acute hypoxia occurrence, but its level begins to reduce in prolonged hypoxia. In chronic hypoxia, an increased level of HIF-2α is observed [2]. Regulation of this switch from the HIF-1α isoform to the HIF-2α isoform includes several mechanisms. First, the HIF-2α isoform seems more resistant to the hydroxylation maintained by PHDs and FIH and regulations by other factors; hence it shows much higher stability than HIF-1α [12,13]. The HIF-2α transcriptional activity promotes the activation of the hypoxia-associated factor (HAF) whose activity leads to the ubiquitination of the HIF-1α subunit resulting in its degradation [14]. Other possible mechanisms in hypoxia-inducible factors switch are based on the anti-sense RNA expression activated by the HIF-2α, chromatin modifications, activity of the response element 1-silencing transcription factor (REST), and miRNA regulations [8].

In physiological circumstances, the hypoxia-inducible factor takes part in embryonic development, circulatory system development, chondrogenesis, adipogenesis, osteogenesis, hematopoiesis, and the development of the immune system elements. It plays a protective role in coronary artery disease, peripheral arterial disease, wound healing, organ transplant rejection, and colitis. However, the HIF factor also contributes to the pathogenesis of hereditary erythrocytosis, traumatic shock, pulmonary diseases, sleep apnea, and cancers [5].

## 3. Activation of the Hypoxia-Inducible Factor in Cancers

The state of hypoxia is the characteristic feature of solid tumors. Existent blood vessels support the growth of cancer cells until the tumor mass exceeds 2 mm^3^. After that, tumor growth relies on the oxygen supply provided by the new blood vessels developed in the process of angiogenesis. However, vascularization often leads to the formation of disorganized or leaky vessels, which result in creating hypoxic or even anoxic areas [15]. Nonetheless, the correlation between the occurrence of hypoxia regions and upregulation of the hypoxia-inducible factor is non-consistent, suggesting other underlying mechanisms of its activation. Furthermore, malignancies originating from hematopoietic cells that do not form tumors also show upregulation of the hypoxia-inducible factor [7]. A significant percentage (88%) of normal human tissues does not contain HIF-1α due to its quick degradation. Still, it can be detected in over half of the cancers, such as prostate cancer, breast cancer (BC), lung cancer, pancreatic cancer, brain cancer, stomach cancer, ovarian cancer, kidney cancer, and melanomas [16]. Constitutive expression of the hypoxia-inducible factor is also observed in non-hypoxic cancer cell lines [17]. The level of the HIF-1α negatively correlates with the degree of cell differentiation, proving that more undifferentiated and less mature and specialized cells, like cancer cells, tend to contain more HIF-1α [18].

The hypoxia-inducible factor activation can be induced in various ways, not limited to the result of hypoxia occurrence. One of the simplest explanations is a mutation in gene coding of the von Hippel-Lindau suppressor protein. Alteration in the *VHL* gene is the earliest event during the development of renal cell carcinoma which is the most common type of kidney cancer. The von Hippel-Lindau suppressor mutation results in constant expression of the alpha subunit of the HIF despite the oxygenation level [19]. The presence of reactive oxygen species common in cancers can also activate the hypoxia-inducible factor by creating a pseudohypoxic state. An elevated level of ROS can potentially damage the cell but sublethal load stimulates the HIF transcriptional activity and impairs the activity of prolyl hydroxylases [15]. Therefore, the decreased ROS production is associated with a reduced level of the alpha subunit [20]. Another feature of cancer cells that can result in HIF activation is the dysregulation of transduction pathways. Overactivation of signaling pathways PI3K/PTEN/Akt, RAS/RAF/MAPK, and NFκB, due to activation through cytokines, chemokines, G protein-coupled receptors, and toll-like receptors, can also modulate the HIF signaling [4]. Activation of the transcriptional factor can also result from an increased level of oncometabolites—canonical metabolites present in cells that accumulated above a certain level possess pro-oncogenic properties. Succinate, fumarate, pyruvate, and lactate can allosterically inhibit PHDs resulting in stabilizing the alpha subunit [21]. In triple-negative breast cancer, an increase in HIF-1α level is caused by excessive glutamate secretion [4]. Another alteration in the prolyl hydroxylases activity is caused by the low availability of 2-oxoglutarate or α-ketoglutarate, which are substrates of PHDs, or by deficiency of iron, whose binding to the catalytic center is required for the enzymatic activity of hydroxylases [7,21]. Increased glycolysis in cancers, associated with the Warburg Effect, also contributes to the activity of the hypoxia-inducible factor activation, and the factor’s activity contributes to glucose metabolism. The phenomenon results in the increased level of pyruvate dehydrogenase kinase (PDK) which inhibits prolyl hydroxylases creating a feedback loop, in which the increased activity of the HIF causes an increase in the transcriptional factor level [19]. Increased activity of the M2 isoform of pyruvate kinase, often associated with the Warburg effect, can also contribute to the HIF activation. Besides that, increased lactate production, one of the most characteristic features of aerobic glycolysis, also stabilizes the alpha subunit by inhibiting PHDs [7]. Less extensively discussed factors, whose activity contributes to the hypoxia-inducible factor stabilization, include the decreased activity of the p53, factors modulating the VHLp activity (c-Myc, WSB1), mTOR mediated gamma rays stimulation of stabilization, and changes in calcium homeostasis [21].

In breast cancer, like in other types of cancer, the HIF overexpression origin is not limited to the hypoxia itself. In a 3D model of MCF7-HER2 culture, an increased level of HIF-2α was reported to be present outside the hypoxic regions of spheroids [22]. Breast cancers present several unique ways of non-canonical hypoxia-inducible factors activity induction. For example, increased activity of the PI3K/Akt/mTOR pathway observed in breast cancer can lead to an increased level of HIF-1α. Although increased signal transduction pathways activity is not exclusive to this type of cancer, in BC, overexpression of HER2 or ER receptors may contribute to an increased level of HIF-α through the PI3K/Akt/mTOR pathway [23]. Moreover, an increased level of an ER receptor enables an increase in HIF-1α levels through the estrogen response elements located within the *HIF1A* promoter [15]. ER-α directly induces HIF-1α transcription, but ER-β has an opposite effect on HIF activity by degrading HIF-1β, hence deteriorating the dimerization of subunits [24]. The ER-β2, a splicing variant of the receptor ER-β, is involved in the regulation of PHD3, which subsequently results in HIF-α stabilization. Although triple-negative breast cancers lack the ER-α and full-length splicing variant of ER-β, the ER-β1, and ER-β2 were reported to be expressed in the triple-negative breast cancer cell line MDA-MB-231 [25]. Despite the relation between estrogen receptors and the hypoxia-inducible factor, based on the immunohistochemistry of the clinical samples of breast cancer, a higher frequency of HIF-1α was observed in the ER-α negative samples compared to the ER-α positive one [26]. The discrepancy between HIFs’ activity and transcript level was already reported in triple-negative breast cancer cells, which may suggest the role of the post-transcriptional regulation mechanisms [15]. The correlation between *HIF2A* expression and ER status was not observed [27]; similarly, the HER2 status depending on *HIF1A* upregulation was not reported [28]. However, a study on clinical samples of breast cancer showed that HIF-2α was significantly higher in the samples with overexpressed HER2 receptors. These findings suggest that HIF-2α, but not HIF-1α, may be regulated by HER2 [22].

Another regulation of HIFs’ activity involves a zinc finger MYND-type containing an 8 (ZMYND8) epigenetic reader. Its role presents a positive feedback loop mechanism, in which ZMYND8 interacts physically with an α subunit of HIF and elevates its activity. Increased transcriptional activity of HIFs, both HIF-1α, and HIF-2α, leads to the upregulation of ZMYND8, closing the loop. ZMYND8 upregulation and a role in the progression and metastasis of breast cancer were reported to be associated with the activity of the hypoxia-inducible factors [29]. Extracellular ATP can also drive HIFs’ activation in normoxia via the Akt pathway [30]. The activity of this transduction pathway, induced by the presence of the extracellular ATP, results in increased activity of PGK1, which forms a feedforward loop with the HIF-1α isoform, whose activity leads to the upregulation of PGK1 [31]. PGK1 also complexes with the HIF-2α isoform boosting its activity [32]. Another element interacting directly with an α subunit is frequently overexpressed in breast cancer chromodomain helicase DNA binding protein 4. CHD4′s interaction with HIF-1α and HIF-2α increases the recruitment of HIF and RNA Pol II, which results in exceeding the expression of HIFs’ downstream genes both in normoxia and hypoxia [33]. Tumor-associated macrophages also play a considerable role in activating hypoxia-inducible factors. For example, the transmission of long non-coding RNA stabilizing HIF-1α under normoxia in breast cancer cell line MDA-MB-231 was reported. HISLA, for HIF1α-stabilizing long non-coding RNA, interacts with PHD2, deteriorating its role in the degradation of the α subunit of the transcriptional factor. The impact on the HIF-2α isoform has not yet been investigated [34]. The activity of the breast cancer-associated macrophages leads to the suppression of succinate dehydrogenase, which results in the accumulation of the oncometabolite succinate. An increase in succinate level then causes the inhibition of PHDs, hence the stabilization of an α subunit of HIFs [35]. PHDs’ activity can also be altered by their substrates deficiency. A study on breast cancer cell lines, 4T1 and MDA-MB-231, showed that mitochondrial malic enzyme 2 might decrease the level of α-ketoglutarate, leading to HIF-1α stabilization [36].

As it was already mentioned in the review done by de Heer et al., high activity of HIFs and low levels of their mRNA level at the same time are often reported, especially in triple-negative breast cancer. Those discrepancies may result from many non-canonical induction pathways observed in breast cancers and perhaps post-transcriptional regulation mechanisms that need further investigation [15]. Moreover, the HIF-3α regulatory activity on other isoforms remains heavily unexplored. Some splicing variants of the HIF-3α isoforms are believed to inhibit HIF-1α and HIF-2α isoforms [8,37].

## 4. Effects of the HIF Activation

The earliest discovered role of the hypoxia-inducible factor was stimulating the formation of new blood cells by inducing the activity of the erythropoietin gene [38]. Over the last two decades of intensive research, it turned out that the function of the HIF factor exceeds far beyond. In the state of insufficient oxygenation, protein synthesis gets limited only to elements crucial for survival, but given the plurality of non-canonical regulation mechanisms observed in cancers, especially breast cancers, the role of hypoxia-inducible factors seems much more complex.

### 4.1. Metabolism

One of the most intensively researched effects of the hypoxia-inducible factor activity on cancer cells’ metabolism is its role in suppressing oxidative phosphorylation and maintaining glycolysis, which in cancers, can manifest as the Warburg Effect. The occurrence of hypoxia or activation of the HIF factor does not affect the number of mitochondria. Still, the factor seems to orchestrate a shift from OXPHOS (oxidative phosphorylation) toward increased glycolytic metabolism and significant changes in mitochondrial metabolism. These changes enable maintaining energy production and redox balance [39]. The HIF-1α elevates the pyruvate dehydrogenase kinase activity, preventing pyruvate conversion to acetyl-CoA by inhibiting pyruvate dehydrogenase (PDH), resulting in a decreased flux of pyruvate into the TCA (tricarboxylic acid) cycle. The second activity of the factor limiting substrate flows into the Krebs cycle is increasing the lactate dehydrogenase activity, often overexpressed in cancers, which converts pyruvate into lactate [20]. The hypoxia-inducible factor decreases the oxidative capacity of mitochondria and reduces their activity by inducing the activity of less effective components of the electron transport chain, stimulates NADPH production, which regulates mitochondrial redox, and reduces the generation and metabolism of acetyl-CoA. Possibly, the factor also takes part in the expression of a modified, truncated version of voltage-dependent anion channel 1 (VDAC1-ΔC), which is an essential element of metabolic communication between mitochondria and the rest of the cell [37]. Another process inhibited by the hypoxia-inducible factor is the ꞵ-oxidation of fatty acids. Lipolysis inhibition leads to further limitation of the acetyl-CoA, thus decreasing the TCA cycle and resulting in the accumulation of lipid droplets. In cancers, the uptake and synthesis of fatty acids are increased due to the high demand associated with membrane formation, signaling, post-transcriptional modifications, and energy supply [40]. Inhibition of oxidative phosphorylation and ꞵ-oxidation of fatty acids prevents the excessive production of reactive oxygen species and enables maintaining redox balance [41].

Increased glucose metabolism is crucial for the growth and proliferation of cancer cells which require a significant amount of biomass and an alternative energy supply compensating for the impaired OXPHOS. The HIF factor activity is believed to be strongly involved in glucose metabolism, especially the Warburg effect, the phenomenon of glycolysis dependency even in physiological oxygenation. Genes transcribed under the hypoxia-inducible factor activation codes key elements of glucose metabolism, including glucose transporters (GLUT1, GLUT3), enzymes of glycolytic pathways (hexokinases, phosphofructokinase, aldolase, glyceraldehyde 3-phosphate dehydrogenase, phosphoglycerate kinase, enolase, pyruvate kinase, lactate dehydrogenase), and pyruvate dehydrogenase kinase (PDK) [41]. Some of these elements simply allow increased glucose metabolism, but some of them play different roles as well. For example, the M2 isoform of the pyruvate kinase, which is under the regulation of the hypoxia-inducible factor, enables redirecting glycolytic intermediates to other metabolic pathways and stimulates the increased transcriptional activity of the HIF factor by binding to the transactivation domain [42]. Another feedback loop associated with the hypoxia-inducible factor activity is based on the increased level of PDK. As already mentioned, it inhibits prolyl hydroxylases, which causes increased HIF stabilization supporting increased glycolysis [19].

Different effects of the hypoxia-inducible factor activation on metabolism can be observed depending on the isoform. Unlike HIF-1α, HIF-2α tends to lower the level of pyruvate dehydrogenase kinase, which leads to a more oxidative phenotype [19]. The 2α isoform is also involved in glutaminolysis which provides ATP production compensation in reduced production from OXPHOS or glycolysis [37]. A difference can also be seen in the type of the target genes of the factor. Although some gene pools overlap for both isoforms, they have different perforations and unique targets. The HIF-1α activity is more likely to regulate genes associated with glycolysis than HIF-2α [43]. It is believed that some of the exclusive targets of the 1α are genes coding GLUT1, PGK, aldolase, HKII, and PKM2 [44]. Still, some papers report that GLUT1 is a shared target of both isoforms [10] or that, depending on the type of cancer, GLUT1 can be the unique target of HIF-2α, just like in renal cell carcinoma [19].

Studies on HIFs’ activity on metabolism in breast cancer gathered vague conclusions. The main overexpressed isoform in breast cancers is HIF-1α which is believed to be more involved in glucose metabolism [4]. Based on tissue sample studies, HIF-1α is expressed in most normal breast tissue, and its level is elevated in all triple-negative breast cancer samples [28]. A survey of the breast cancer cell lines MCF-7 and MDA-MB-231 revealed that hyperinsulinemia, not hyperglycemia, drives the activity of the HIF-1α rather than the one-way regulatory mechanism between HIF and glucose [45]. One of the most exhaustive studies investigating the activity of HIFs isoforms’ influence on cancer cell metabolism under normoxia was the research done by Bharti et al. in 2018. The clue of the study was to inspect the results of silencing HIF-1α, HIF-2α, or both isoforms in triple-negative breast cancer cell line MDA-MB-231 xenografts. Analysis with magnetic resonance spectroscopy identified no significant changes in pyruvate, glucose, or lactate flux following silencing of HIF-1α, despite the role in glucose metabolism usually assigned to this isoform. Glutathione level increased after HIF-2α silencing but decreased after silencing both isoforms. Silencing the HIF-1α isoform resulted in the reduction of aspartate levels, but silencing HIF-2α elevated it. Only silencing both isoforms simultaneously developed a significant shift in the metabolome, decreasing alanine, aspartate, glycine, tyrosine, acetate, fumarate, pyruvate, glutathione, myoinositol, and uracil [46]. In another study on the impact of tumor-associated macrophage activity on breast cancer cell lines (MDA-MB-231, MDA-MB-468, BT-474, MCF-7), silencing the HIF-1α isoform resulted in reduced glucose uptake and lactate production. The decrease was also observed in the expression of metabolic enzymes. However, it’s difficult to conclude whether those effects are an isolated consequence of HIF-1α silencing, considering the complex interaction with tumor-associated macrophages as well as other isoforms that were not investigated [34].

### 4.2. Evasion of the Immune Response

To survive, cancers have to actively avoid the immune response. There are multiple mechanisms allowing evasion, and the hypoxia-inducible factor plays a crucial role. The HIF factor enhances the expression of CD47 immunoglobulin, which provides a “do not eat me” signal, suppresses the activity of the acquired immune system, and inhibits T-cell proliferation and activation by recruitment of myeloid-derived suppressor cells [41]. In human or murine triple-negative breast cancer cells treated with carboplatin, doxorubicin, gemcitabine, or paclitaxel, expression of CD47, CD73, and PDL1 changed in a HIF-dependent manner and consequently, increase in the ability of cancer cells to evade innate and adaptive immunity was observed [47]. It has also been found that in human TNBC (triple-negative breast cancer) cell lines, the PD-L1 mRNA expression was increased due to augmentation of HIF-1α expression dependent on endoplasmic reticulum oxidoreductase 1-α (ERO1-α). PD-L1-mediated T cell apoptosis was reduced significantly by the knockdown of *ERO1A*. This provides a lead for therapeutic modulation of hypoxia-mediated immunoresistance [48]. The immune response inhibition is also achieved through HIF-induced activation of TGF-ꞵ, VEGF, and chemokine CCL28 [4]. The hypoxia-inducible factor contributes to the acidification of the extracellular environment by modulating the expression of the monocarboxylate transporter 4 (MCT4). Lower pH not only decreases the efficiency of immune response but may also affect the effectiveness of many anti-cancer drugs [44]. Anhydrase 9 plays a critical role in maintaining pH levels. In normal breast tissue, the expression of CA IX is not detected, but it is often expressed in breast cancer [49]. It may be caused by overexpression of HIF-1α, which targets the anhydrase’s gene. It is believed that only the HIF-1α isoform contributes to acidification [50]. This isoform was also reported to activate the multidrug resistance gene (*MDR1*), which codes a pump that actively removes xenobiotics from cells [4]. On the other hand, in a study on MCF-7 mammospheres treated with paclitaxel, the viability of cells was higher after 48-h hypoxia, suggesting a significant role of the HIF-2α isoform in developing chemoresistance [51].

### 4.3. Cell Survival and Death

The hypoxia-inducible factor targets many genes crucial for cancer cell survival. It regulates the cell cycle by targeting cyclin D, cell division through the division cycle-associated protein (CDCA) family, as well as growth and proliferation through VEGF, an insulin-like growth factor 2 (IGF-2), transforming growth factor-alpha (TGF-α), PDGF, and SCF [37]. The factor also modulates the activity of the tumor suppressors, such as p53, proto-oncogenes, such as c-Myc, and critical elements of cellular signalings, such as mTOR [43]. However, depending on the isoform, various effects can be observed. For example, HIF-1α tends to inhibit c-Myc by competitive binding to the transcriptional factor Sp1, while HIF-2α promotes c-Myc activity by enhancing its association with Sp1 [10]. Nonetheless, clinical data on genes expression in breast cancer shows c-Myc overexpression despite the presence of isoform HIF-1α [27].

The hypoxia-inducible factor also regulates apoptosis and autophagy, but the results of experiments investigating those regulations are inconsistent. Autophagy and apoptosis are two mechanisms of programmed cell death. They can be alternatively used by cells to compensate each other if one of the mechanisms is inhibited. However, autophagy is usually upregulated in advanced tumors to help cancer cells adapt to limited nutrients and oxygen conditions. It has been found that HIF-1α and HIF-2α have different impacts on the viability of HepG2 cells grown in spheroid cultures. Cells with HIF-1α knock-down showed increased expression of anti-apoptotic Bcl-XL protein and decreased expression of Bax, which is a pro-apoptotic member of the Bcl-2 family. Tumors lacking HIF-2α develop a survival advantage by enhancing autophagy [52]. Moreover, some types of autophagy are dependent on one HIF isoform; for example, HIF-2α, but not HIF-1α, is a negative regulator of peroxisome abundance in hepatocytes via pexophagy promotion in mice model [53]. In breast cancer, the relationship between isoforms in the regulation of autophagy is not known. Most available data are restricted to HIF-1α. In MCF7 cells exposed to radiation, HIF-1α plays a role in the induction of autophagy by suppression of the PI3K/AKT/mTOR/p70 pathway [54]. Breast cancer cells, MDA-MB-231 treated with paclitaxel showed HIF-1α dependent, down- or up-regulation of the expression of several pro-and anti-apoptotic genes. HIF-1α caused decreased expression of pro-apoptotic genes, i.e., *BAK1*, *CASP3*, *CASP8*, *CASP10*, and *TNFRSF10A* in hypoxia when compared to normoxic conditions in paclitaxel-dependent manner [55]. HIF-1α also caused an increase in Mcl-1 and BNIP-3 expressions. Mcl-1 (myeloid cell leukemia sequence 1) is an anti-apoptotic protein belonging to the Bcl-2 family, which is involved in heterodimerization and neutralization of pro-apoptotic proteins such as Bim or Bax that, as a consequence, suppresses cytochrome c release from mitochondria [56]. BNIP3 is a member of the Bcl-2 family of mitochondrial proteins that activate selective mitochondrial autophagy by binding to Bcl-2 and freeing Beclin-1, which can initiate autophagy [57]. As autophagy and apoptosis may compensate for each other, stimulation of autophagy might be especially important for induction cell death in apoptosis-resistant breast cancer cell lines. Forty-five percent to 75% of breast tumors do not have caspase three protein thus, autophagic death induction is a good alternative when Bcl-2 is inhibited [58].

### 4.4. Angiogenesis, Invasion, and Metastasis

Existing blood vessels support tumor growth to a certain point, beyond which providing oxygen and nutrients requires forming the new vessels. Vascularization of cancer mass is provided in the process of angiogenesis. The activity of the hypoxia-inducible factor takes part in the regulation of genes coding elements essential for formatting new blood vessels: vascular endothelial growth factor (VEGF), stromal cell-derived factor (SDF), angiopoietin (ANGPT), placental growth factor (PGF), platelet-derived growth factor (PDGF), and stem cell factor (SCF) [41]. All of these factors are regulated by HIF-1α, but in some cases, the regulation by HIF-2α has also been proved. HIFs’ target genes, whose products play a role in angiogenesis, invasion, and metastasis, are presented in Table 1. Gene categories containing only the HIF-1α isoform do not exclude the role of the HIF-2α in regulation. Studies described in included articles often did not investigate HIF-2α regulation.

The type of the target genes, for example, coding erythropoietin, nitric oxide synthases, or endothelin-1, suggests that the HIF factor not only takes part in the formation of new blood vessels but also regulates blood parameters [42]. Angiogenesis can be inhibited by some splicing variants of the HIF-3α, which hinder the HIF/VEGF pathway through complexing with the alpha subunit [37]. Although the HIF-1α is the leading isoform in breast cancer, the HIF-2α isoform is a critical regulator of physiological and pathophysiological angiogenesis and, at least, similarly important to HIF-1α [89].

According to the earliest estimates, metastases are responsible for almost two-thirds of cancer-related deaths, and this number increases up to three-quarters in the case of breast cancer [90]. Metastasis is the multistep process enabling cancers to spread to adjacent or distant tissues. The hypoxia-inducible factor regulates every step of this process; modification of extracellular matrix, conversion from epithelial to mesenchymal phenotype (EMT), detachment from the basement membrane, invasion of surrounding tissues or passage into blood or lymph vessels, avoidance of immune response and anoikis, passage out of vessels, preparing niches for colonization in new tissue, and transition from mesenchymal to epithelial phenotype [41]. The HIF factor regulates the metastatic process through interaction with the *TWIST1* gene, inhibition of E-cadherin, regulating ꞵ-catenin pathway, and enhancing expression of *ZEB1*, *SNAI1*, and SNAI2 (SLUG) [32,37,85,91]. In breast cancer, EMT, migration, and invasion are implemented by Notch signaling. The gain-of-function mutation has not been observed in breast cancer, so dysregulation is most probably caused by other regulation mechanisms. Both HIF-1α and HIF-2α isoforms affect Notch signaling, although the HIF-2α isoform seems to have a more potent impact [92]. Silencing HIF-2α in breast cancer cell line MDA-MB-231 resulted in the expression of the epithelial phenotype [32]. HIF-1α and HIF-2α factors activate the expression of MMPs (MMP1, −2, −9, −14) that participate in the degradation of extracellular matrix components and degrade the basement membrane, which results in easy migration and spread of cancer cells [32,79,80,81,82,83]. CXC chemokine receptor 4 (CXCR4) and 3 (CXCR3) were found to be expressed in many tumors including breast and significantly correlated with invasion, angiogenesis, metastasis, and prognosis. In breast cancer, HIF-1 dependent expression of these receptors increases the migration of cells and increases their potential to survive in the circulation stage [67,68,69]. HIF-1α and HIF-2α have some overlapping functions, but they also have unique and sometimes even opposing effects. Interesting results were obtained by Todd et al. concerning the role of HIF-1α and HIF-2α isoforms in metastasis of breast cancer. Using the transgenic mouse models of mammary carcinoma with tumor-specific deletion of *HIF1A* and *HIF2A* let to evaluate the effects of HIF modulation on tumor dissemination to multiple distant sites. It has been found that deletion of *HIF1A* slows down primary tumor growth, and decreases the spreading of cancer cells to the bones while increasing lung metastasis. Conversely, *HIF2A* in the primary tumor forces tumor cells to spread to the bone but is dispensable for their dissemination to the lung [69].

HIF signaling plays a role in cell extravasation by affecting the expression of the gene that encodes for L1 cell adhesion molecule involved in breast cancer cell adhesion to endothelial cells, as well as *ANGPTL4* coding for angiopoietin-like 4 that decreases adhesion between endothelial cells [62]. HIFs also affect the expression of factors that prepare the lung microenvironment for metastatic colonization, such as LOX and LOX-like proteins (LOXL2 and LOXL4) [32,75,76,77].

Recent studies suggest that expressions of some long noncoding RNAs that are HIF-dependent may participate in the metastatic phenotype of breast cancer cells. In breast cancer cells, the lncRNA RAB11B-AS1 is transcriptionally induced in hypoxia by HIF-2α. RAB11B-AS1 enhances hypoxia-induced VEGFA and ANGPTL4 expression in breast cancer and promotes angiogenesis and distant breast cancer metastasis in mice [74]. In hypoxia, the lncRNA BCRT1 is transcriptionally regulated by HIF-1α and facilitates EMT [72]. The studies of Gómez-Maldonado et al. showed HIF-mediated transcriptional upregulation of lncRNAs encoded by the *EFNA3* locus. Using animal models and in vitro assays, they demonstrated that Ephrin-A3 expression leads to metastatic spread [73]. Liu et al. identified HIF-1α as a potential transcription factor of lncRNA *HCG18*. There was a positive correlation between *HCG18* and HIF-1α expression in breast cancer tissues. Moreover, knockdown of HIF-1α suppressed *HCG18* expression in breast cancer cells. It has been found that HIF-1α binds to specific HREs in the promoter region of *HCG18* and promotes HCG18 expression. In vivo assays revealed that reducing *HCG18* expression in MDA-MB-231 cells caused inhibition of tumor growth and lung metastasis in xenograft mouse models. *HCG18* by miR-103a-3p binding positively regulates the expression UBE2O (ubiquitin conjugating enzyme E2O) and thus activates the UBE2O/mTORC1 axis in breast cancer cells [70].

### 4.5. Cancer Stem Cells

A significant part of human cancers may originate from stem cells or early progenitor cells that transform into cancer cells with stem cell properties [44]. The hypoxia-inducible factor plays a crucial role in generating, maintaining, and gaining malignant features by cancer stem cells. The HIF factor activity regulates stemness through interaction with genes like *POU5F1* (October-4 protein), *SOX2*, *NANOG*, KLF4 (Krüppel like factor 4) [44,93]. HIF-1α transcriptionally upregulates the expression of CD47, which helps to escape the phagocytosis of macrophages and maintain the stem phenotype of breast CSCs (cancer stem cells) [94]. In various cancers, including breast, hypoxia can promote the induction and development of CSCs through CD44 and aldehyde dehydrogenase [95]. High activity of ALDH is associated with, self-renewal of CSCs, metastasis, tumorigenesis, and poor prognosis in breast cancer [96]. HIF-1α can induce the expression of *ALDH1A1* in breast cancer cells under hypoxic conditions [97]. Interestingly, it was shown that expression *of ALDH1A1* was also associated with HIF-2α expression in breast cancer cell lines and samples. Moreover, ALDH inhibitor suppressed HIF-2α expression and self-renewal in BCSCs derived from 4T1 cells [98].

It has been demonstrated that the upregulation of *ITGA6* coding for integrin alpha 6 by HIF modulates stem cell-like features and invasion of MDA-MB-231 breast cancer cells [99]. HIF-1α can also be recruited by TAZ to the promoter of *CTGF* encoding of connective tissue growth factor, which is the key factor in promoting the stem-like phenotype of breast CSCs [100]. HIFs play a critical role in chemotherapy-induced BCSC enrichment by up-regulation of dual-specificity phosphatases (DUSP9 and DUSP16) [101]. DUSP9 and DUSP16 dependent inactivation of ERK and stimulation of p38 led to upregulation of Nanog and KLF4, which are key transcription factors of stem cells [102].

### 4.6. HIFs Activity and Hormone Receptors

The exclusive effect of HIFs’ activity on breast cancers is their association and influence on hormone receptors. Estrogens are essential for breast cancer growth and development. They affect estrogen response elements whose activation leads to the expression or inhibition of target genes [103]. In the breast cancer, hypoxia induces the expression of around 1000 genes, but only 42 share dependency on HIFs’ activity. Among them, 9 demonstrate different expression patterns in ER-positive subtypes compared to ER-negative subtypes, and three of that gene group contain binding sites for both ER and HIF [65]. It was observed that hypoxia inhibits ER expression in ER-positive breast cancers. In the study on ER-positive breast cancer cell lines, hypoxia reduced ER-α levels by 75% and ER-β by 50%. However, inducing hypoxia in the ER-negative breast cancer line MDA-MB-231 resulted in the induction of ER-α expression. In ER-positive breast cancer cell lines, T47D and MCF7 knockdown of HIF-1α resulted in decreased ER-α and increased ER-β levels, although the second one got reduced after 48 h of hypoxia. These findings suggest a potential role of HIFs in maintaining the ER-α/ER-β ratio and point out the necessity of investigating the role of HIFs’ isoforms in hormone receptors regulation [103].

## 5. Perspectives on HIF’s Inhibition in Breast Cancer—To Inhibit or Not to Inhibit?

The hypoxia-induced factors’ level correlates with a few breast cancer characteristics. Although, earlier studies did not confirm the correlation between HIF-1α expression and clinicopathological characteristics such as molecular type, receptors status, tumor size, or age [28,104], correlation with lymph node status, and higher expression level of HIF-1α in samples of bigger tumor size has been reported [105]. Moreover, loss of the HIF-1α isoform was reported to be correlated with better treatment outcomes [104], and HIF-1α positivity was associated with worse prognostics [26]. In the meta-analysis done by Shamis et al., a high level of HIF-1α showed a correlation with poorer OS (overall survival) and DFS (disease-free survival) [106]. The HIF-1α has been recognized as a potential marker of neoadjuvant chemotherapy response marker [107] and bone metastasis marker [69]. The relationship between tamoxifen therapy and the level of HIFs is noteworthy as well. Tamoxifen treatment increases HIF-1α positivity in breast cancers [26] and prevents the downregulation of the HIF-2α expression [24].

In the case of the HIF-2α isoform, its high expression seems to be linked with better overall survival, but in HER-positive breast cancer patients [24]. Other possible correlations of this isoform’s expression require further investigation.

**Figure 1:**
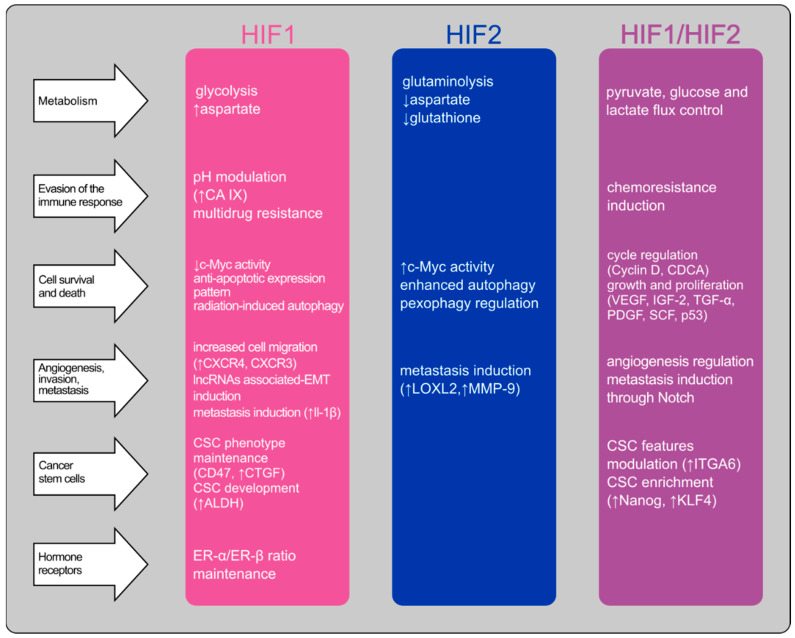
The potential roles of hypoxia-inducible factor isoforms in breast cancer biology. The scheme summarizes the impact of the hypoxia-inducible factors’ activity in breast cancer with a particular focus on the differences between HIFs isoforms. The right column includes the effects of both isoforms’ activity or ones that cannot be assigned to a specific isoform due to a lack of adequate investigation. ↑↓ Increased or decreased expression, activity or amount.

The results of the HIFs inhibition can be pre-checked using silencing or deletion methods in breast cancer cell lines studies. Knocking down the HIF-1α isoform in triple-negative breast cancer cell line (MDA-MB-231) showed no effect on proliferation, level of apoptosis-related proteins survival, Bcl-2 or Bak, and hypoxia-induced chemoresistance [108,109]. The outcome of the HIF-1α downregulation in MDA-MB-231 was inconsistent with the results in another triple-negative breast cancer cell line, SUM149 [109]. Repressing HIF-1α could effectively restore proliferation and invasiveness in MCF-7 cancer cells previously treated with the antidiabetic drug. However, the same study showed that HIF-1α inhibition could decrease chemoresistance in diabetic breast cancer patients [45], the used inhibitor was PX-478 which presented only some specificity to the HIF-1α isoform [110]. HIF-2α silencing resulted in decreased ATP-driven invasion [32], mammosphere formation, and an increased number of apoptotic cells under hypoxia conditions [111]. The HIF-2α isoform displays an intriguing relation with receptor status in breast cancer. HER-2 overexpressing breast cancers are considered to be more prone to HIF-2α inhibition [22], and estradiol (E2) downregulates HIF-2α, but only in ER-positive breast cancer cells [24]. Discrepancies can also be observed in the results of HIFs’ isoforms’ inhibition of metastasis. Deletion of HIF-1α decreased primary tumor formation and bone metastasis but increased the total tumor burden and lung metastasis. Deleting the HIF-2α isoform reduced metastasis to the bone but not to the lung [69]. On the other hand, knocking mitochondrial malic enzyme 2, which inhibited HIF-1α stabilization, inhibited lung metastasis, gathering different conclusions from the previously mentioned study [36]. One of the most exciting results was drawn from a survey on HIFs stabilization in breast cancer cell lines. Treating MDA-MB-231 breast cancer cells with a PHD inhibitor, molidustad, which stabilized hypoxia-inducible factors, resulted in decreased viability, growth, and clone formation, induced cell cycle arrest, and chemosensitivity. These findings suggest that depending on the molecular context, HIFs may be endowed with anticancer activity [112]. The role of HIF isoforms in breast cancer is summarized in Figure 1.

As de Heer points out, targeting HIFs in breast cancer therapy has been ineffective so far [12]. However, questioning whether HIFs were even targeted in therapy is justified. So far, agents that are claimed to alternate the activity of those factors, do not appear to target HIFs directly but seize the effect through the more global mechanism of action. Moreover, most of them target only HIF-1α, or rather: it is the only interaction that has been investigated. Most of the comprehensive HIFs inhibitors affect the activity of topoisomerase (CPT11, SN-38, EZN-2208), mTOR pathway (Miconazole, Temsirolimus, Sirolimus, Everolimus), HSP90 (17-AAG, 17-DMAG, IPI-504, Geldanamycin, Ganetespib), or other elements indirectly associated with HIFs’ regulation. In opposition to the inhibitors mentioned so far, a certain group of inhibitors targets hypoxia-inducible factors directly. PT2399, PT2385, and PT2977 are synthetic compounds that bind directly to the HIF-2α preventing dimerization, halting HIF activity in the hypoxia response elements [113,114]. PT2977 seems to be the most promising agent in this group, and it is currently tested beyond its initial model of renal cancer (NCT02974738, NCT04924075). Although data gathered during the first clinical trials of these compounds is encouraging, it has not been tested in breast cancer yet.

The lack of effective breast cancer therapy based on HIFs inhibitors and the elusive role of those factors in this type of cancer raises the concern whether targeting hypoxia-inducible factors is the right path to take. Results of the study on breast cancer cell lines suggest the need to consider aspects like HIF-1α versus HIF-2α inhibition, double versus singular isoform inhibition, different hormone receptors status, metastases, and perhaps different not yet investigated issues. In other words, targeting hypoxia-inducible factors in breast cancers should be preceded by a better understanding of their role in this type of cancer.

## 6. Conclusions

The lack of effective breast cancer therapy based on HIFs inhibitors and the elusive role of those factors in this type of cancer raises the concern that targeting hypoxia-inducible factors is the right path to take. Results of the study on breast cancer cell lines suggest the need to consider aspects like HIF-1α versus HIF-2α inhibition, double versus singular isoform inhibition, different hormone receptors status, metastases, and perhaps different not yet investigated issues. In other words, targeting hypoxia-inducible factors in breast cancers should be preceded by a better understanding of their role in this type of cancer.

## Figures and Tables

**Table 1:** Target genes of the hypoxia-inducible factors and their role in angiogenesis, invasion, and metastasis.

Target Gene	HIF Isoform Regulation	Protein/RNA Role	References
A2BAR	HIF-1α	adenosine receptor 2A signaling in breast cancer cells promotes filopodia formation, invasion, and metastasis	[59]
ADAM12	HIF-1α and HIF-2α	disintegrin and metalloproteinase 12 (ADAM12); trim off the extracellular domain of the membrane-bound heparin-binding epidermal growth factor-like growth factor (HB-EGF), which binds to EGFR and triggers a signal transduction pathway involved in metastasis	[60]
AMF	HIF-1α	autocrine motility factor; stimulates motility of cells under hypoxia	[61]
ANGPTL4	HIF-1α	angiopoietin-like 4; a secreted factor that inhibits EC-EC interaction	[62]
BRK	HIF-1α and HIF-2α	Breast tumor kinase, increases migration via modulation of EMT -associated molecules	[63]
CALR	HIF-1α	Calreticulin; facilitates invasion and metastasis by promoting the breast cancer stem cell phenotype through Wnt/β-catenin signaling	[64]
CASP14	HIF-1α	Caspase 14; breast cancer stem cell marker; has also been implicated as a gene found in brain-metastatic compared to non-brain metastatic breast cancer	[65]
CKB	HIF-1α	Creatine kinase brain isoform, is a major effector of the HIF-1α-mediated promotion of metastatic phenotypes in ER-negative breast cancer	[66]
CXCR3	HIF-1α	CXCR3 is a Gαi G-protein coupled receptor (GPCR), a seven transmembrane spanning receptor; is the receptor for CXCL10 chemokine, increases migration	[67]
CXCR4	HIF-1α	CXCR-4 is an alpha-chemokine receptor specific for stromal-derived-factor-1 (SDF-1 also called CXCL12); tumor cells overexpressing CXCR4, have higher potential to survive the circulation stage	[68,69]
HCG18	HIF-1α	Long Noncoding RNA HLA complex group 18; positively regulates the expression of BC-related ubiquitin-conjugating enzyme E2O (UBE2O) by sponging miR-103a-3p, thus promoting the malignant phenotypes of BC cells through the UBE2O/AMPKα2/mTORC1 axis.	[70]
ITGA5	HIF-1α and HIF-2α	integrin that binds to fibronectin, promotes lung metastasis in orthotopic transplantation models of triple negative breast cancer	[71]
L1CAM	HIF-1α	L1 cell adhesion molecule; integral membrane glycoprotein belonging to a large class of immunoglobulin superfamily cell adhesion molecules (CAMs) that mediate cell-to-cell adhesion at the cell surface	[62]
LncRNA BCRT1	HIF-1α	Long Noncoding RNA (breast cancer-related transcript 1; promotes cell mobility and tumor metastasis in breast cancer; regulate the EMT process	[72]
LncRNA EFNA3	HIF-1α	Long Noncoding RNA ephrin A3, increases protein ephrin 3 levels by sequestering miRNAs away from EFNA3 mRNA allowing for its translation;	[73]
LncRNA RAB11B-AS1	HIF-2α	Long Noncoding RNA RAB11B-AS1; increases the expression of angiogenic factors including VEGFA and ANGPTL4 in hypoxic breast cancer cells through the recruitment of RNA Pol II	[74]
LOX	HIF-1α	Lysyl Oxidase, post-translationally modifies collagen molecules in the extracellular matrix (ECM), regulates cell adhesion, motility and invasion; increases metastasis by enhancing premetastatic niche formation	[75,76,77]
LOXL2	HIF-1α and HIF-2α	lysyl oxidase-like 2; post-translationally modifies collagen which is a component of the extracellular matrix (ECM); promotes lung metastasis by facilitating the formation of the pre-metastatic niche	[32,77]
MAFF	HIF-1α	v-maf musculoaponeurotic fibrosarcoma oncogene homolog F; transcription factor regulating tumor invasion and metastasis via IL11/STAT3 pathways activation;	[78]
MMP1	HIF-2α	degradation of the extracellular matrix MMP-1, mostly type I collagen, promotes tumor growth and metastasis particularly to the brain	[79]
MMP2	HIF-1α and HIF-2α	matrix metalloproteinase 2; degradation of extracellular matrix; degrades type IV collagen	[80,81,82]
MMP9	HIF-1α and HIF-2α	matrix metallopeptidase 9; degradation of the extracellular matrix	[32,83]
MMP14/MT1-MMP	HIF-1α and HIF-2α	matrix metalloproteinase-14 or MT1-MMP, is a member of the membrane-type MMP subfamily; degradation of the extracellular matrix	[79]
P4HA1	HIF-1α and HIF-2α	prolyl 4-hydroxylase-α1; essential for collagen biogenesis; 4-hydroxyproline residues are necessary for the proper folding of collagen polypeptide chains into stable triple helical molecule; HIF-1α-dependent ECM remodeling increases cell motility and promote invasion and metastasis.	[79]
P4HA2	HIF-1α	prolyl 4-hydroxylase-α2; essential for collagen biogenesis; 4-hydroxyproline residues are necessary for the proper folding of collagen polypeptide chains into a stable triple helical molecule; HIF-1α-dependent ECM remodeling increases cell motility and promotes invasion and metastasis.	[79]
PDGFB	HIF-1α	platelet-derived growth factor B; promotes, metastasis of hypoxic breast cancer cells via lymphatic dissemination	[84]
PGF	HIF-1α	placental growth factor, promotes the metastasis of breast cancer cells; promotes recruitment of mesenchymal stem cell, to the primary tumor site and, stimulates them to the expression of CXCL10	[67]
SNAIL1	HIF-2α	transcriptional factor; plays a key role in the control of epithelial to mesenchymal transition	[32]
SLUG	HIF-1α	transcriptional factor; plays a key role in the control of epithelial to mesenchymal transition	[85],
TWIST	HIF-1α	transcriptional factor; plays a key role in the control of epithelial to mesenchymal transition	[86]
VEGF	HIF-1α	vascular endothelial growth factor; stimulates the formation of blood vessels	[87,88]
